# Transparent and flexible Sb-doped SnO_2_ films with a nanoscale AgTi alloyed interlayer for heat generation and shielding applications

**DOI:** 10.1039/c7ra12988b

**Published:** 2018-01-10

**Authors:** Kyung-Su Cho, Han-Ki Kim

**Affiliations:** Kyung Hee University, Department of Advanced Materials Engineering for Information and Electronics 1 Seocheon Yongin Gyeonggi-do 446-701 Republic of Korea imdlhkkim@khu.ac.kr +82-31-205-2462 +82-31-201-2462

## Abstract

Transparent and flexible Sb-doped SnO_2_ (ATO) films with a nanoscale AgTi alloyed interlayer were fabricated for use as plasma damage-free, indium-free, thermally stable electrodes for high performance heat generating films and shielding films in smart windows. The AgTi alloy-inserted ATO film on a PET substrate showed a low sheet resistance of 6.91 ohm per square and a high optical transmittance of 90.24% without thermal annealing or intentional substrate heating. Even after deformation using an outer bending radius of 4 mm, the ATO film with a AgTi interlayer showed a constant sheet resistance due to the mechanical robustness of the AgTi interlayer. Furthermore, the AgTi-inserted ATO film showed a constant resistance even after annealing at 500 °C, unlike the Ag-inserted ATO films. Furthermore, we demonstrated the feasibility of the AgTi-inserted ATO films as transparent heat generating films and shielding films for smart windows. The effective heat generation and shield performance of the ATO/Ag–Ti/ATO multilayer suggests that the multi-functional ATO/Ag–Ti/ATO films can be used to create energy-efficient smart windows for building energy management systems and automobiles.

## Abstract 2

Transparent and flexible ATO films with a nanoscale AgTi alloyed interlayer were fabricated for high performance heat generating and shielding films in smart windows.

## Graphical abstract

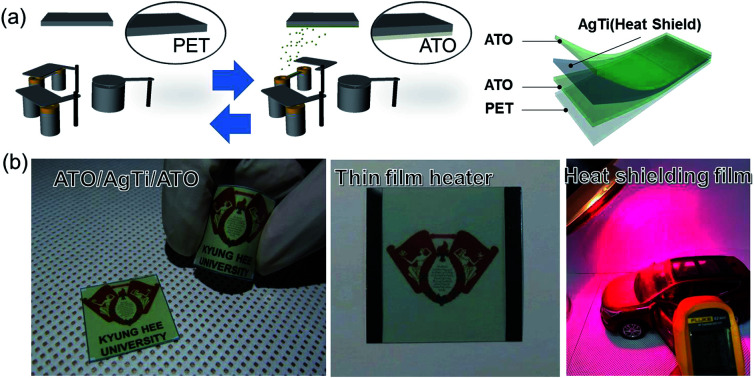

## 1 Introduction

The rapid development of building energy management systems (BEMSs) requires high performance heat generating films and heat shielding film-based smart windows.^1–5^ In addition, automobiles require high performance heat generating and heat shielding windows for removing ice or frost on the window and blocking sunlight from outside. Metal-based semi-transparent films coated on flexible substrates or glass substrates have been employed to fabricate simultaneous heat generating and heat shielding films.^6,7^ Sputtered or evaporated Ag films on flexible substrates have mainly been used because Ag-based films generate high temperatures through resistive heating by obtaining power from heat absorbed at near infrared (NIR) wavelengths. However, Ag-based heat generating and shielding films have very low optical transmittance due to the inherent opacity of the metal film.^7,8^ Therefore, it is important to develop highly transparent and flexible metal-based electrodes for heat generating and shielding films to replace conventional Ag-based semitransparent electrodes. Due to the absence of metal-based transparent electrodes, Ag-based semi-transparent electrodes were applied in heat shielding films, and a Sn-doped In_2_O_3_ (ITO) film was applied in a heat generating film.^7,9–11^ Metallic transparent electrodes with metallic conductivity, high optical transmittance comparable to ITO films, and outstanding mechanical flexibility are required to realize heat generating and shielding films for smart windows. Although various transparent electrode materials films have been reported as transparent electrodes for thin film heaters, such as conducting polymers, carbon (carbon nanotubes, graphene, graphene oxide), Ag nanowires, Cu nanowire, metal meshes, metal network, and oxide–metal–oxide, there are no reports of metallic transparent electrodes that can simultaneously be used in thin film heat generating and heat shielding films.^12–34^ Recently, we reported semi-transparent Ag–Cu electrodes that could be applicable in thin film heaters and heat shielding films simultaneously, but the low optical transmittance of Ag–Cu alloy film remained a critical problem.^7^

In this study, we investigated the electrical, optical, morphological, and mechanical properties of ATO films on PET substrates with a thermally evaporated Ag–Ti alloy layer for use as transparent, flexible, thermally stable electrodes for heat generating and shielding films in smart windows. Based on figure of merit values, the optimal ATO thickness was determined for highly transparent and conductive electrodes. In addition, the mechanical flexibility of the Ag–Ti-inserted ATO films were tested using specially designed bending testers. Furthermore, we demonstrated the feasibility of thermally evaporated ATO/Ag–Ti/ATO films for high performance simultaneous heat generating films and heat shielding films.

## 2 Experimental

### 2.1 Thermal evaporation of ATO/Ag–Ti/ATO films

**Fig. 1:**
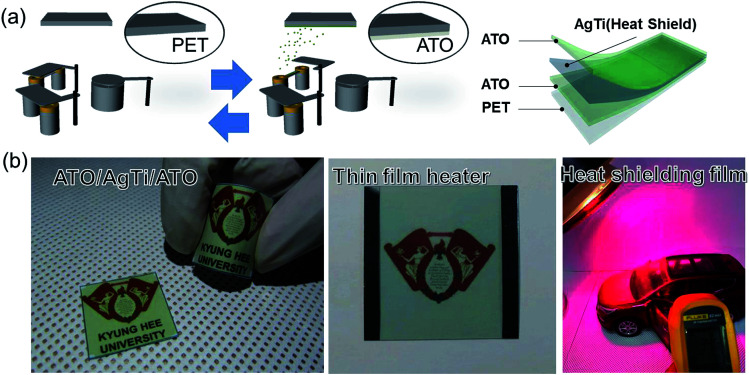
(a) Schematic of the thermal evaporation process and structure of ATO/Ag–Ti/ATO multilayer. (b) Picture of transparent and flexible ATO/Ag–Ti/ATO multilayer coated on PET substrate. The Kyung Hee University symbol can be clearly seen behind the curved sample due to its high optical transmittance (above 90%) and good flexibility. The remaining pictures show promising applications for the ATO/Ag–Ti/ATO film in thin film heater and heat shielding film applications.

Highly transparent and flexible ATO/Ag–Ti/ATO films were thermally evaporated on PET substrates at room temperature without intentional substrate heating using a thermal evaporation system (NNS Vacuum 15NNS005). The ATO powder source and 95 wt% Ag 5 wt% Ti alloy source were loaded in a tungsten boat, and the evaporation chamber was evacuated to 1 × 10^−6^ torr. First, the bottom ATO layer was thermally evaporated on a PET substrate at a voltage of 0.48 V, a current of 53 A, a *Z*-factor of 0.724, and a tool factor of 128%. During thermal evaporation of the ATO film, the PET substrate was constantly rotated at a speed of 10 rpm. Then, the metallic Ag–Ti interlayer was continuously evaporated onto the bottom ATO layer at a voltage of 0.34 V, a current of 55 A, a *Z*-factor of 0.529, and a tool factor of 155%. After evaporation of the Ag–Ti alloy interlayer, the top ATO layer was finally evaporated on the Ag–Ti interlayer at the same evaporation conditions as the bottom ATO layer. The continuous evaporation process is illustrated in Fig. 1a, and a picture of the optimized transparent and flexible ATO/Ag–Ti/ATO sample is shown in Fig. 1b. The thickness of the ATO and Ag–Ti layers was precisely controlled using a thickness monitor in the thermal evaporation system.

### 2.2 Characterization of the ATO/Ag–Ti/ATO films

The electrical properties of the thermal evaporated ATO/Ag–Ti/ATO films were examined using Hall measurements (HL5500PC, Accent Optical Technology) as a function of ATO thickness. The optical transmittance of the ATO/Ag–Ti/ATO multilayer was measured using a UV/visible spectrometer (UV 540, Unicam). The thermal stability of Ag–Ti and Ag interlayers in ATO films was evaluated by comparing the sheet resistance after rapid thermal annealing from 300 to 600 °C. The resistance change in the ATO/Ag–Ti/ATO multilayer was measured during inner/outer bending and twisting tests to demonstrate the flexibility of the multilayer. In addition, dynamic fatigue bending at a fixed bending radius of 10 mm and twisting tests at a fixed twisting angle of 15° were performed using a lab-made cyclic bending and twist test machine operated at a frequency of 1 Hz for 100 000 cycles. Field emission scanning electron microscopy (FESEM) was employed to investigate the surface morphology of the ATO/Ag–Ti/ATO multilayers before and after bending tests. In addition, microstructure and interface of the ATO/Ag–Ti/ATO multilayer were investigated by transmission electron microscopy (TEM) examination. The TEM sample was prepared by cutting of sample, polishing, and ion milling with liquid nitrogen cooling.

### 2.3 Fabrication and evaluation of heat generating films and heat shielding films

To demonstrate that thermally evaporated ATO/Ag–Ti/ATO multilayers can be used for flexible and transparent heat generating and shielding films, we fabricated a typical thin film heater with a size of 25 × 25 mm^2^ and a heat shielding film with a size of 80 × 80 mm^2^ (Fig. 1b). Two terminal Ag contact electrodes were sputtered on the edge of the ATO/Ag–Ti/ATO multilayer for ATO/Ag–Ti/ATO-based thin film heaters. A DC voltage was supplied by a power supply (OPS 3010, ODA technologies) to the ATO/Ag–Ti/ATO films through an Ag contact electrode at the film edge. The temperature of ATO/Ag–Ti/ATO-based heater was measured using a thermocouple mounted on the surfaces of the transparent electrode and an IR thermal imager (A35sc, FLIR). The heat shielding performance of the ATO/Ag–Ti/ATO multilayer was examined using a halogen lamp that irradiated the ATO/Ag–Ti/ATO multilayer while the temperature inside an automobile (shown in Fig. 1b) was measured. An IR thermometer (62-MAX, Fluke) was used to measure the inside temperature of a model automobile with a bare PET window and an ATO/Ag–Ti/ATO/PET window. The ATO/Ag–Ti/ATO multilayers used as a thin film heater and a heat shielding film were identical samples prepared at the same evaporation conditions.

## 3 Results and discussion

**Fig. 2:**
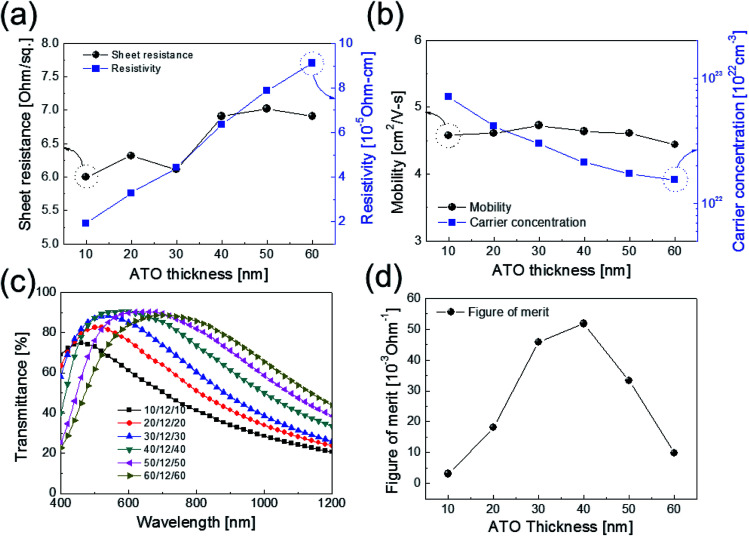
(a) Sheet resistance, resistivity, (b) carrier mobility, and concentration of thermally evaporated ATO/Ag–Ti/ATO multilayer as a function of top and bottom ATO thickness at a fixed Ag–Ti interlayer thickness of 12 nm. (c) Optical transmittance values of ATO/Ag–Ti/ATO multilayers for different top and bottom ATO thicknesses at a constant Ag–Ti thickness of 12 nm. (d) FOM value of the ATO/Ag–Ti/ATO multilayer as a function of ATO thickness.

Fig. 2a and b show the Hall measurement results of the ATO/Ag–Ti/ATO multilayer film as a function of the thickness of the top and bottom ATO layers at a fixed Ag–Ti thickness of 12 nm. The existence of thicker top and bottom ATO layers with high resistivity led to an increase in resistivity of the ATO/Ag–Ti/ATO multilayer film, as shown in Fig. 2a. The increased resistivity could be attributed to the low carrier mobility regardless of ATO thickness as shown in Fig. 2b. The carrier concentration injected from the metal Ag–Ti interlayer decreased due to an increase in the top and bottom ATO layer volume at a constant Ag–Ti thickness of 12 nm. This increased the resistivity of the ATO/Ag–Ti/ATO multilayer film. Fig. 2c shows the optical transmittance of the ATO/Ag–Ti (12 nm)/ATO multilayer film with increasing top and bottom ATO layer thicknesses. The ATO/Ag–Ti/ATO multilayer film with 40 nm-thick top and bottom ATO layers had the highest optical transmittance of 90.24% at a wavelength of 550 nm. The absorption edge of the ATO/Ag–Ti/ATO multilayer shifted to longer wavelengths as the top and bottom ATO layer thicknesses increased. At a fixed Ag–Ti thickness of 12 nm, the optical transmittance and absorption edge of the multilayer are influenced by the top and bottom oxide thicknesses.^34–36^ Although the ATO/Ag–Ti/ATO multilayer showed a high optical transmittance in the visible wavelength region (400–800 nm), the optical transmittance of the ATO/Ag–Ti/ATO multilayer in the near IR region was also low due to the existence of a metallic Ag–Ti interlayer. The low near IR transmittance of the ATO/Ag–Ti/ATO multilayer makes it suitable for use as a heat shielding film to prevent heat flow through a window. Although the ATO film with 14 nm thick Ag–Ti interlayer showed lower sheet resistance (4.82–6.93 ohm per square) regardless of the ATO thickness, an increase in Ag–Ti interlayer thickness led to reduction of optical transmittance (87.83%). To apply ATO/Ag–Ti/ATO multilayer as transparent electrode, higher optical transmittance is very important. Therefore, we selected the optimal thickness of Ag–Ti interlayer as 12 nm. As shown in Fig. 2d, figure of merit (FOM) values were calculated as a function of the thickness of the top and bottom ATO layers based on the sheet resistance and optical transmittance at a wavelength of 550 nm for the ATO/Ag–Ti/ATO multilayer. The ATO/Ag–Ti/ATO multilayer film with a 40 nm-thick ATO layer exhibited the highest FOM value of 51.83 × 10^−3^ ohm^−1^ due to a low sheet resistance of 6.91 ohm per square and a high optical transmittance of 90.24% at a wavelength of 550 nm. Therefore, we determined that the optimal thicknesses of ATO and the Ag–Ti layer were 40 and 12 nm, respectively.

**Fig. 3:**
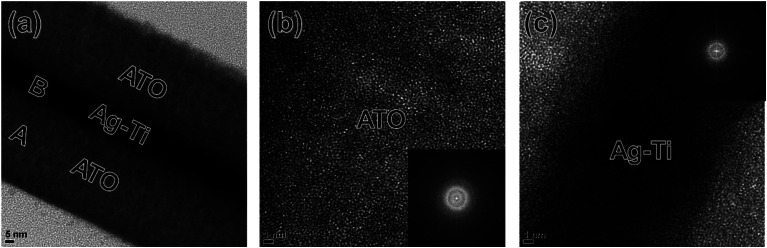
(a) Cross-sectional TEM image of the thermally evaporated ATO/Ag–Ti/ATO multilayer film on a PET substrate. Enlarged TEM images obtained from A and B region in the cross-sectional image with an inset of FFT patterns; (b) bottom ATO region (region A) and (c) Ag–Ti interlayer region (region B), respectively.

To investigate microstructure of thermal evaporated ATO and Ag–Ti interlayer, TEM examination was carried out. Fig. 3a showed a cross-sectional TEM image of the ATO/Ag–Ti/ATO multilayer grown on PET substrate. Due to exact thickness control during thermal evaporation, the multilayer showed symmetric structure with clearly distinguished layers. It was clearly shown that the metallic Ag–Ti interlayer connected the top ATO and bottom ATO layer and acts as main conduction path. Because the thermal evaporation process was carried out at room temperature, the multilayer had well-distinguished interlayer without interfacial layer. The enlarged TEM image in Fig. 3b indicates the bottom ATO obtained from “A” region in the cross-sectional TEM image. The thermal evaporated ATO layer showed a typical amorphous structure with short-range-order. In addition, diffuse fast Fourier transform (FFT) pattern in the inset of Fig. 3b indicated that thermal evaporated ATO layer had a typical amorphous structure unlike conventional FTO or ATO films grown by chemical vapor deposition. Fig. 3c showed enlarged Ag–Ti interlayer obtained from “B” region in the cross-sectional TEM image. As indicated FFT pattern in the inset of Fig. 3c, the thermal evaporated Ag–Ti layer showed a crystalline structure even though it was prepared at room temperature.

**Fig. 4:**
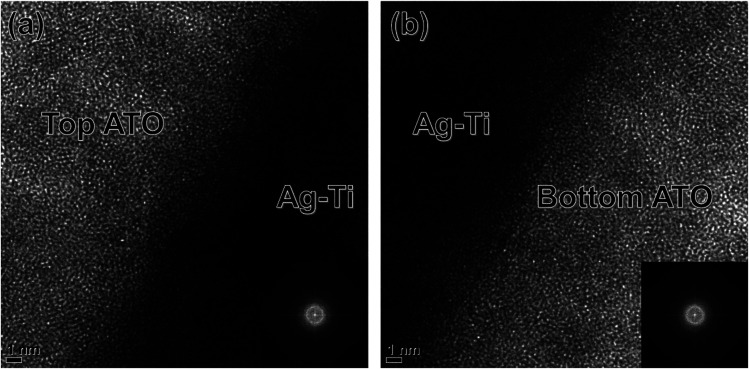
Enlarged TEM images obtained from (a) top ATO/Ag–Ti interface and (b) Ag–Ti/bottom ATO interface region with an inset of FFT patterns.

Fig. 4 showed enlarged TEM images obtained from the interface region between amorphous ATO layer and crystalline Ag–Ti interlayer. Because the thermal evaporation process of bottom ATO, Ag–Ti interlayer, and top ATO layer was continuously conducted without breaking vacuum, there is no interfacial layer between ATO and Ag–Ti interlayer. Both interface (Ag–Ti/bottom ATO and top ATO/Ag–Ti) showed a discrete and well-defined interface between bright amorphous ATO and dark crystalline Ag–Ti interlayer. Unlike sputtered ITO top layer, which is easily crystallized on the crystalline metal interlayer, in sputtered ITO/Ag/ITO multilayer, the evaporated top ATO layer in Fig. 4a showed identical amorphous structure to the bottom ATO layer in Fig. 4b due to low energy of evaporated ATO atoms.^37^

**Fig. 5:**
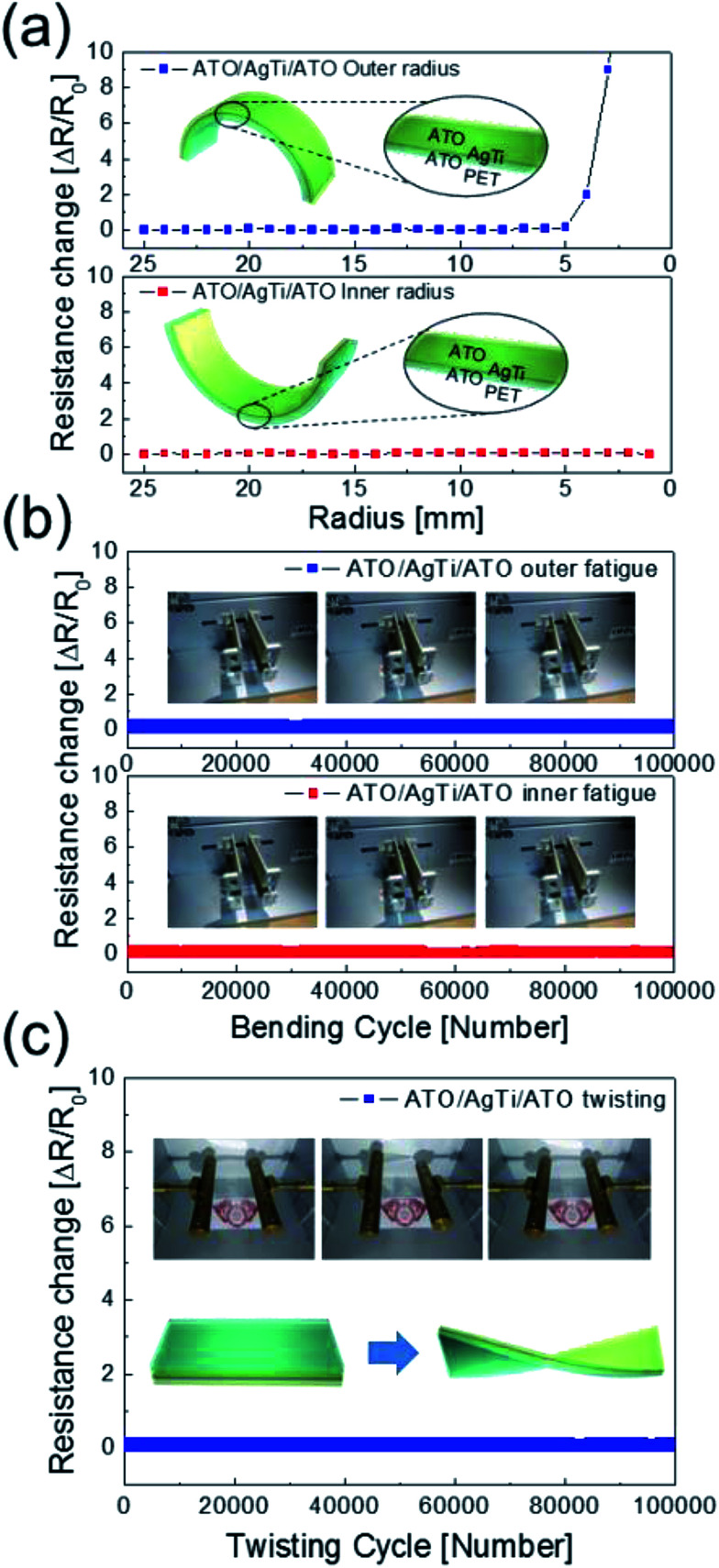
(a) Outer and inner bending test results for the flexible and transparent ATO/Ag–Ti/ATO multilayer with decreasing bending radius. The inset indicates that the outer bend experienced tensile stress, while the inner bend experienced compressive stress. (b) Cyclic outer and inner fatigue test results of the ATO/Ag–Ti/ATO multilayer with increasing bending cycles at a constant bending radius of 10 mm. Inset panels exhibit the outer bending steps during fatigue testing. (c) Twisting test results of the ATO/Ag–Ti/ATO multilayer with increasing twisting cycles at a constant twisting angle of 15°.

The mechanical flexibility of the thermally evaporated optimal ATO/Ag–Ti/ATO multilayer film was investigated using a lab-designed inner and outer bending test system. Fig. 5a shows the results of the outer/inner bending tests for the ATO/Ag–Ti/ATO multilayer electrodes with decreasing outer/inner bending radius. We measured the resistance change (Δ*R*) of the ATO/Ag–Ti/ATO multilayer during substrate outer/inner bending to determine the critical bending radius. *R*_0_ represents the initial resistance, and *R* indicates the measured resistance. The outer/inner bending test result in Fig. 5a shows that the evaporated ATO/Ag–Ti/ATO (40/12/40 nm) multilayer had a constant resistance until the bending radius reached 5 mm. The following equation can be used to calculate the peak strain for a curved ATO/Ag–Ti/ATO multilayer with decreasing bending radius:^38^1a
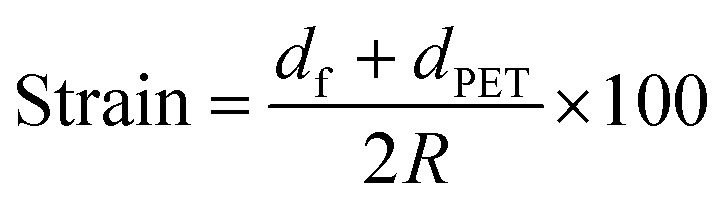
here, *d*_f_ and *d*_PET_ are the thicknesses of the ATO/Ag–Ti/ATO multilayer and the PET substrate, respectively. The ATO/Ag–Ti/ATO multilayer film (40/12/40 nm) on a 125 μm-thick PET substrate experienced a peak strain of 1.25% at a bending radius of 5 mm. The resistance of the ATO/Ag–Ti/ATO multilayer rapidly increased beyond the critical bending radius due to separation of cracked films that experienced tensile stress. In the inner bending tests, the ATO/Ag–Ti/ATO multilayer showed no change in resistance even though cracks formed on the sample. Under the compressive stress illustrated in the inset picture of Fig. 5a, the cracked ATO/Ag–Ti/ATO films overlapped or physically touched. Fig. 5b shows the dynamic outer and inner bending fatigue test results for the ATO/Ag–Ti/ATO multilayer with increasing bending cycles at a fixed bending radius of 10 mm and a repeating rate of 1 Hz. Both dynamic outer and inner bending fatigue tests showed no change in resistance (Δ*R*) during 100 000 bending cycles, demonstrating the good flexibility of the ATO/Ag–Ti/ATO multilayer film. We employed a twisting test as another mechanical flexibility test. The inset pictures of Fig. 5c show images of the lab-designed twisting test steps. Twisting the sample allowed us to investigate the mechanical stability of the transparent electrode. Fig. 5c exhibits the twisting bending test results of the ATO/Ag–Ti/ATO multilayer at a constant twisting angle of 15°. Over 100 000 repeated twisting tests, the ATO/Ag–Ti/ATO multilayer showed no change in resistance due to the outstanding flexibility of ATO/Ag–Ti/ATO multilayer. Overall, the electrical and optical analysis results and the mechanical tests showed that insertion of a metallic Ag–Ti layer into the ATO layer is an effective way to reduce the resistivity and improve the mechanical flexibility of the evaporated ATO films.

**Fig. 6:**
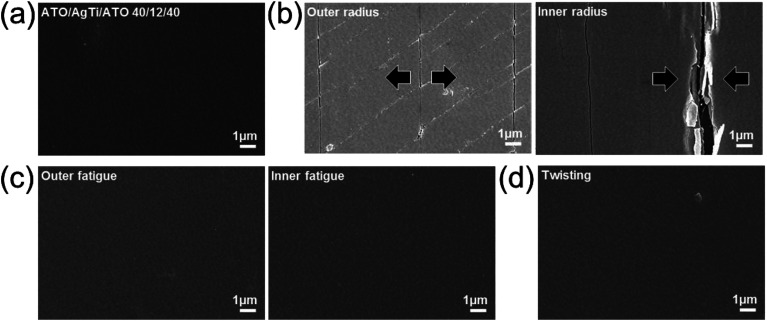
Surface FESEM images of the evaporated ATO/Ag–Ti/ATO multilayer before and after bending tests. Surface FESEM image of (a) as-evaporated ATO/Ag–Ti/ATO and (b) after outer/inner bent ATO/Ag–Ti/ATO film beyond a critical bending radius. Arrows indicate the cracks on the ATO/Ag–Ti/ATO films. Surface FESEM images of ATO/Ag–Ti/ATO films after (c) dynamic fatigue test and (d) twisting test showing identical surface morphologies as the as-evaporated sample.

Fig. 6 shows surface FESEM images of ATO/Ag–Ti/ATO (40/12/40 nm) multilayers before and after outer/inner bending, dynamic fatigue, and twisting tests. Surface FESEM images (Fig. 6a) of as-evaporated ATO/Ag–Ti/ATO films showed a smooth and featureless morphology because the evaporation occurred at room temperature. Fig. 6b shows surface FESEM images of the cracked ATO/Ag–Ti/ATO multilayer after outer and inner bending at a bending radius of 1 mm, which is beyond the critical bending radius. Several cracks were observed on the ATO/Ag–Ti/ATO multilayer because the outer bending test applied severe tensile stress to the films. The enlarged FESEM image shows that the cracks on the sample physically separated the ATO/Ag–Ti/ATO multilayer film and increased the measured resistance change during outer bending. Even though the sample showed no resistance change during inner bending in Fig. 5a, there were also several cracks in the multilayer. Unlike the outer bending test, the cracks of inner bending test have a ‘hill’ near the edge due to overlaps in the cracked region during the inner bending test. This physically overlapped region could conduct current during the inner bending test, so the inner bent sample showed a constant resistance change even though the sample was severely cracked. There were no cracks in the multilayer after the dynamic fatigue tests shown in Fig. 6c and d because they experienced repeated stress at a constant bending radius and angle below the critical bending angle and radius. The ATO/Ag–Ti/ATO multilayer showed a similar surface FESEM image to the as-deposited sample even after 100 000 dynamic outer/inner bending and twisting cycles, as shown in Fig. 6c and d. The outstanding mechanical flexibility of the ATO/Ag–Ti/ATO multilayer indicates that the thermally evaporated ATO/Ag–Ti/ATO multilayer film is a promising flexible and transparent electrode for flexible heat generating films and shielding films used in curved or shaped smart windows.

**Fig. 7:**
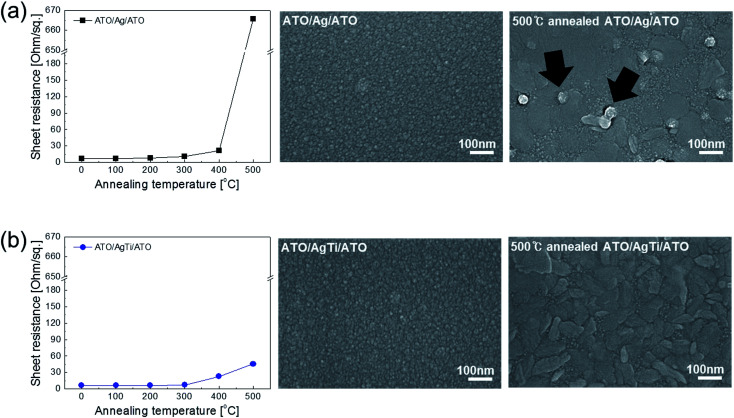
Resistance of (a) ATO/Ag/ATO and (b) ATO/Ag–Ti/ATO multilayers with increasing annealing temperature. The right side shows the surface FESEM images of the ATO/Ag/ATO and ATO/Ag–Ti/ATO before and after 500 °C annealing.

To illustrate the thermal stability of the Ag–Ti inserted ATO film, we compared the resistance change of Ag-inserted ATO and Ag–Ti-inserted ATO films as shown in Fig. 7. The resistance of the Ag-inserted ATO films abruptly increased after rapid thermal annealing at 500 °C. This increased resistance was due to agglomeration and out-diffusion of Ag though the grain boundaries of crystalline ATO layers, as we reported in our previous work.^37^ The surface FESEM image in Fig. 7a showed a crystallized ATO top layer and agglomeration of Ag on the top of the ATO layer. However, the Ag–Ti-inserted ATO film showed a smaller resistance change after rapid thermal annealing at 500 °C due to the thermal stability of the Ag–Ti interlayer, as shown in Fig. 7b. Surface FESEM images also showed only crystallized ATO top layers without agglomeration of the metal layer. The thermal stability of the Ag–Ti-based ATO electrode indicates that the Ag–Ti-inserted ATO is an appropriate electrode for heat generating films.

**Fig. 8:**
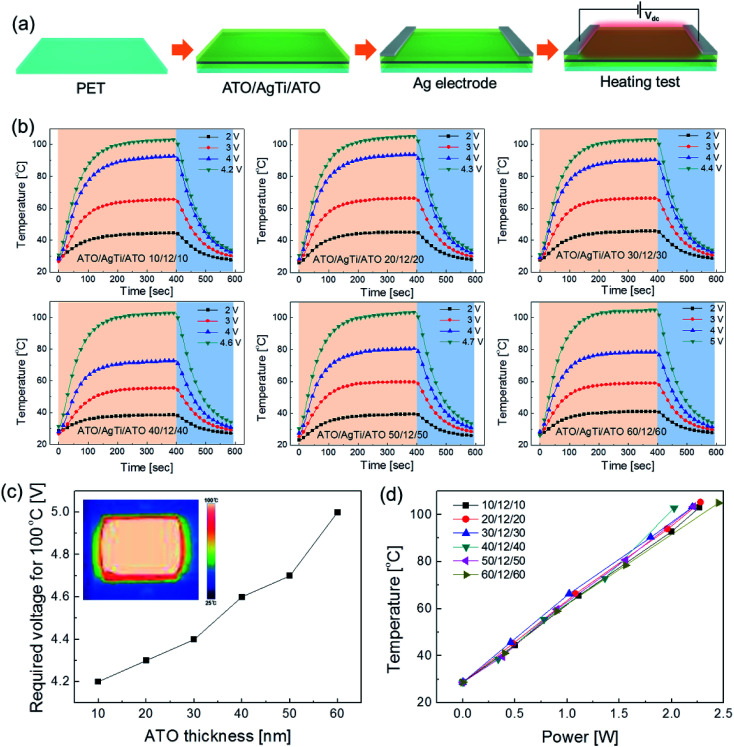
(a) Fabrication process for thin film heaters using ATO/Ag–Ti/ATO electrodes. (b) Temperature plots of thin film heaters with increasing input DC voltage as a function of ATO thickness. (c) Input voltage of ATO/Ag–Ti/ATO thin film heaters to obtain a saturation temperature of 100 °C as function of ATO thickness. (d) Temperature of the thin film heater as a function of input power.

ATO/Ag–Ti/ATO multilayer film-based thin film heaters were fabricated to investigate the feasibility of the thermal evaporated ATO/Ag–Ti/ATO multilayer as a flexible and transparent electrode for heat generating films in smart windows. The fabrication process is illustrated in Fig. 8a. As shown, the ATO/Ag–Ti/ATO-based thin film heater had a two-terminal contact electrode at the edges of the devices. To generate heat in the films, a DC voltage was applied to the thin film heaters by a power supply through an Ag metal contact electrode at the film edge. The temperature of the ATO/Ag–Ti/ATO-based thin film heaters was measured *in situ* using a thermocouple in direct contact with the surface of the thin film heaters. Fig. 8b shows the temperature profiles of the ATO/Ag–Ti/ATO-based thin film heaters at different input DC voltages as a function of ATO layer thickness. When a DC voltage was supplied to the ATO/Ag–Ti/ATO-based thin film heaters, the temperature of the thin film heater rapidly increased and reached a saturation temperature. It is noteworthy that the increase in the ATO layer thickness of the thin film heater increased the DC voltage required to obtain an identical temperature. At an identical input voltage, the ATO/Ag–Ti/ATO-based thin film heater with a thinner ATO layer showed a higher saturation temperature. Fig. 8c shows the DC voltage of the thin film heater required to obtain a temperature of 100 °C with increasing ATO thickness. An increase in ATO layer thickness led to an increase in the DC voltage required to reach the saturation temperature of 100 °C because the thinner ATO-based electrode had a lower sheet resistance. The higher saturation temperature of the thin film heater with a thinner ATO layer indicates efficient transduction of electric energy into Joule heating in ATO/Ag–Ti/ATO electrodes. As we described by Huang *et al.*, the saturation temperature can be can be expressed as:^39^2a
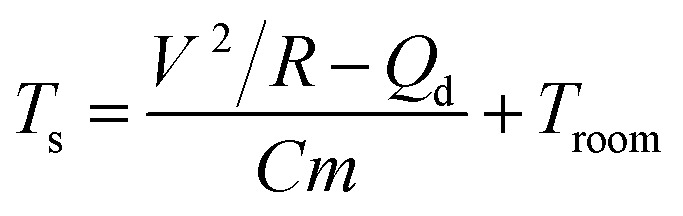
here, *V* is the applied voltage, *R* is the resistance of thin film heater, *Q*_d_ is heat dissipation, *C* is heat capacity of the film, *m* is film mass, and *T*_s_ and *T*_room_ are the saturation and room temperature, respectively. Because main heat dissipation path is air convection, temperature of thin film heater can be saturated at *T*_s_ when Joule heating and air convection reached a dynamic balance.^7,36^ It is apparent from eqn (2a) that the saturation temperature of the thin film heater increases with increasing input DC voltage (*V*) and decreasing resistance (*R*). Therefore, a lower sheet resistance in the ATO/Ag–Ti/ATO electrode with a thinner ATO layer is imperative for fabrication of high performance thin film heaters with a lower DC input voltage. Fig. 8d shows measured temperature of thin film heaters with different ATO thickness as a function of input power. Regardless of the top and bottom ATO thickness, all thin film heaters showed linearly increased temperature with increasing input power.

**Fig. 9:**
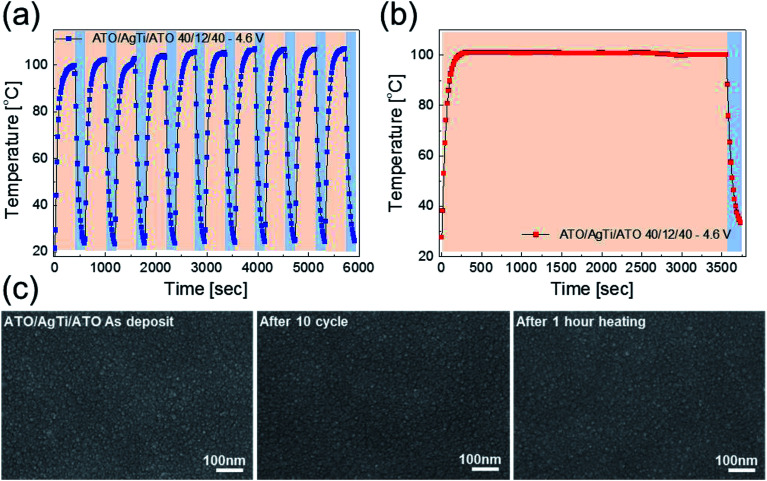
(a) Repeated heating–cooling cycle tests of an ATO/Ag–Ti/ATO-based thin film heater from room temperature to 100 °C. (b) Constant saturation temperature of 100 °C as a function of time to show the thermal stability of ATO/Ag–Ti/ATO electrodes. (c) Surface FESEM images of ATO/Ag–Ti/ATO electrodes in thin film heaters before and after heating.

Fig. 9a shows the repeated heating and cooling profile of the ATO/Ag–Ti/ATO (40/12/40 nm)-based thin film heaters over 10 cycles. The ATO/Ag–Ti/ATO-based thin film heater showed identical heating–cooling profiles and rapidly reached a saturation temperature of 100 °C when a DC voltage of 4.6 V was applied. In addition, when a DC voltage of 4.6 V was supplied to the ATO/Ag–Ti/ATO-based thin film heater for 1 hour, the thin film heater maintained a saturation temperature of 100 °C without temperature modulation, as shown in Fig. 9b. The similar surface FESEM images of the ATO/Ag–Ti/ATO-based thin film heater before and after heating also indicate the durability of the ATO/Ag–Ti/ATO multilayer as an electrode for flexible and transparent thin film heaters.

**Fig. 10:**
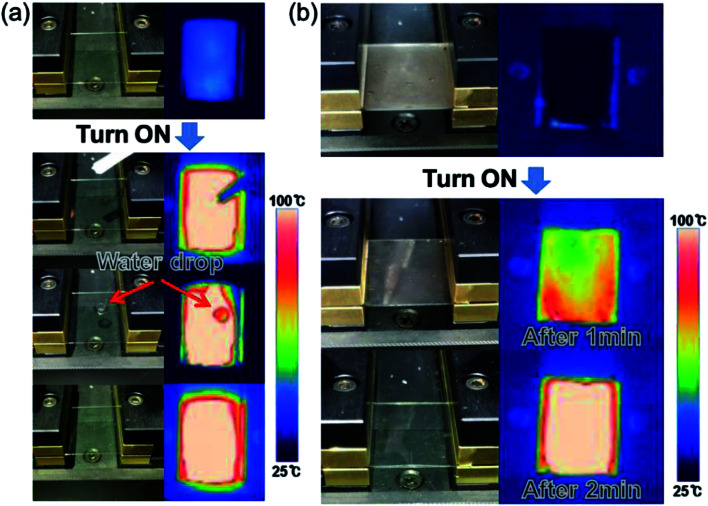
(a) Photo and IR images of the water droplet test on a heated thin film heater with an ATO/Ag–Ti/ATO electrode. (b) Defrosting test results of the ATO/Ag–Ti/ATO-based thin film heater before and after frost formation. The frost on the thin film heater was completely removed by heating the ATO/Ag–Ti/ATO electrode.

A water droplet test was performed to demonstrate the use of ATO/Ag–Ti/ATO-based thin film heaters in smart windows and automobiles. Fig. 10a shows pictures and IR images of the water droplet test of the ATO/Ag–Ti/ATO-based flexible and transparent thin film heater with a saturation temperature of 102.8 °C. When a DC input voltage of 4.6 V was supplied to the thin film heater, a saturation temperature of 102.8 °C was instantly achieved due to a dynamic balance between Joule heating and convection. The IR image indicates the uniformly heated ATO/Ag–Ti/ATO-based thin film heater surface. At the saturation temperature, the water droplet disappeared immediately due to the high temperature of the ATO/Ag–Ti/ATO-based thin film heater. This result demonstrated that ATO/Ag–Ti/ATO-based thin film heaters can be used as self-warming windows. Fig. 10b shows the defrost test of the ATO/Ag–Ti/ATO-based thin film heater before and after frost formation. At an operating DC voltage of 4.6 V, the frost on the surface of the ATO/Ag–Ti/ATO electrode completely disappeared. Therefore, the ATO/Ag–Ti/ATO-based thin film heater could be a transparent and flexible defrost film for automobiles.

**Fig. 11:**
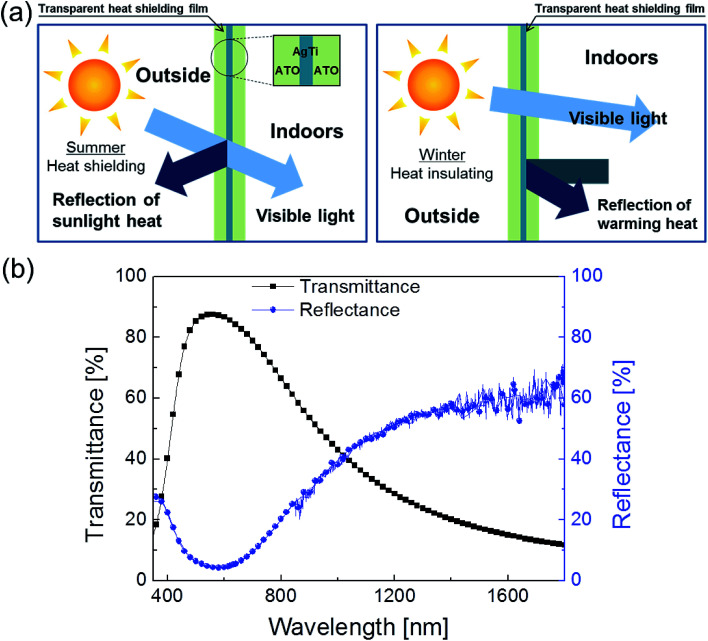
(a) Solar shading and heat insulation through the ATO/Ag–Ti/ATO multilayer on the smart window. (b) Optical transmittance and reflectance of optimized ATO/Ag–Ti/ATO multilayer.

Because solar shading and heat shielding windows are need in smart buildings and automobiles, smart windows must transmit visible light and shield IR radiation. To enable smart windows to balance the transmittance and sunlight-energy-shielding, it is necessary to form cut-off filters that transmit visible light and reflect near-infrared (NIR) light, as shown in Fig. 11a. Moreover, because heat shielding on the indoor side of the window is produced by reflecting far-infrared light, which is a warming radiant heat energy, the reflection of far-infrared light is also required. Fig. 11b shows the transmission and reflection spectrum of the ATO/Ag–Ti/ATO multilayer in the visible to the near-infrared light region. The multilayer shows high optical transmittance in the visible wavelength region and high reflection in the NIR wavelength region, which is appropriate for heat shielding films. The ATO/Ag–Ti/ATO multilayer ensures good lighting with 90% visible light transmittance and high solar shading. The total energy consumption in a building or automobile can be effectively controlled by effective shielding of heat though a ATO/Ag–Ti/ATO film attached to a smart window.

**Fig. 12:**
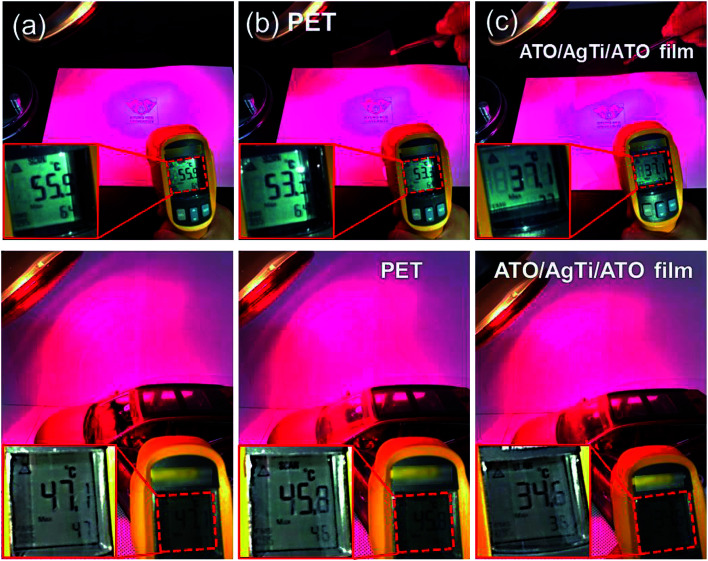
Photographs demonstrate the heat-shielding performance of the ATO/Ag–Ti/ATO multilayer on a glass substrate and automobile model. Irradiation of a halogen lamp through (a) air, (b) bare PET substrate, and (c) ATO/Ag–Ti/ATO-coated PET film and *in situ* measured temperature.

Fig. 12a and b shows a heat shielding test of a bare PET substrate and an ATO/Ag–Ti/ATO multilayer-coated PET, respectively. Irradiating glass with light from a halogen lamp resulted in an increase in temperature. The surface temperature of the glass was measured *in situ* using a portable IR thermometer. The surface of the glass without the heat shielding film showed a temperature of 55.9 °C under halogen lamp irradiation as shown in Fig. 12a. When the light was blocked by a bare PET substrate, there was a small decrease in temperature (53.3 °C). However, when the radiation was blocked by a transparent ATO/Ag–Ti/ATO multilayer film, the surface temperature of the glass substrate was significantly reduced from 55.9 to 37.1 °C. Inside the automobile model, the temperature was 47.1 °C under halogen lamp irradiation without heat shielding film (shown in Fig. 12b). When the light was blocked by bare PET in a front window, there was a small decrease in temperature (45.8 °C). However, when the radiation was blocked by a transparent ATO/Ag–Ti/ATO multilayer in the front window, the indoor temperature of the car was significantly reduced from 47.1 °C to 34.6 °C, indicating that the ATO/Ag–Ti/ATO multilayer was effective in heat shielding due to its high reflection in the NIR region. Therefore, the thermally evaporated ATO/Ag–Ti/ATO multilayer can be used as a heat generating and heat shielding film simultaneously in automobile windows or smart windows for BEMS.

## 4 Conclusions

We developed thermally evaporated ATO/Ag–Ti/ATO multilayers for use as flexible and transparent films for transparent and flexible heat generating films and heat shielding films. To optimize the thickness of ATO film in the multilayer, the effect of ATO thickness on the sheet resistance and optical transmittance of the ATO/Ag–Ti/ATO films was examined. Based on FOM values calculated from the sheet resistance and optical transmittance of the ATO/Ag–Ti/ATO multilayer, we obtained an optimized ATO/Ag–Ti/ATO multilayer film with a sheet resistance of 6.91 ohm per square and an optical transmittance of 90.24%. In addition, the thermally evaporated ATO/Ag–Ti/ATO electrode showed a small critical outer/inner bending radius and outstanding flexibility. Moreover, the thermal stability of the Ag–Ti interlayer was compared with a typical Ag interlayer. The time-dependent temperature profile of the thin film heater with the ATO/Ag–Ti/ATO electrodes demonstrated that the thermally evaporated multilayer is a promising transparent electrode for high performance thin film heaters for smart windows and automobiles. Furthermore, we investigated the heat shielding performance of the ATO/Ag–Ti/ATO multilayer for use as an energy efficient solar shading film on smart window and automobiles. The simultaneous heat generation and heat shield performance of the optimized ATO/Ag–Ti/ATO multilayer indicated that it is feasible to use this ATO/Ag–Ti/ATO multilayer to create energy-efficient automobile windows and smart windows for BEMS.

## Conflicts of interest

There are no conflicts to declare.

## Supplementary Material
